# 

**Published:** 1845-03

**Authors:** 


					) Vol. V,
MARCH, 1845.
No. I>
THE ,
AMERICAN
JOURNAL
AND
LIBRARY
OF
EDeutal
0 ciettct.
PUBLISHED
QUARTERLY
PUBLISHED UNDER THE AUSPICES OP THE
American Sottctg of Cental Sutfltons.
editors:
CHAPIN A. HARRIS M. D., D. D. S.
EDWARD MAYNARD, M. D., D. D.S.
AMOS WESTCOTT, M.D.D.D. S.
BALTIMORE:
ARMSTRONG 8c BERRY.
JOHN W. WOODS, NWfltBR.
$5 00 per annnntrp?raAlein admnce.
This number contains 7 sheets.

				

